# GDF15 (Growth/Differentiation Factor-15) Expression in Human Adipose Tissue and in Adipocyte Cell Lines

**DOI:** 10.3390/biomedicines14061329

**Published:** 2026-06-11

**Authors:** Emily Wilfurth, Alexandra Höpfinger, Edita Islami, Thomas Karrasch, Andreas Schäffler, Andreas Schmid

**Affiliations:** Department of Internal Medicine III, Giessen University Hospital, 35392 Giessen, Germany

**Keywords:** GDF15, adipocyte, adipose tissue, adipokine, 3T3-L1, Toll-like receptor, hypoxia

## Abstract

**Background**: GDF15 (growth/differentiation factor-15) is part of the transforming growth factor-beta family and represents a cellular stress-induced gene. It might have a role in metaflammation and adipoflammation. We aimed to investigate the effects of Toll-like receptor (TLR) activation and hypoxia-related pathways together with metabolic factors on GDF15 regulation in adipocytes and adipose tissue (AT). **Methods**: GDF15 mRNA quantities in the human adipocyte cell line SGBS, in visceral (VAT) and subcutaneous adipose tissue (SAT) (resected from n = 96 obese and characterized patients), and in murine 3T3-L1 adipocytes were measured by real-time RT-PCR. GDF15 protein concentrations in cell supernatants and serum were quantified by ELISA. The following stimuli/pathways were investigated: insulin, glucose, TLR ligands (TLR2/6, TLR3, TLR4, TLR7, TLR9), bile acids, synthetic FXR/TGR5 activators, and HIF1α activators. **Results**: Basal GDF15 expression is low and only marginally induced in SGBS cells. In contrast, GDF15 is expressed in human SAT and VAT and correlates positively with the corresponding GDF15 protein concentration in peripheral blood serum of obese patients. Among metabolic factors, insulin and bile acids such as ursodeoxycholic acid upregulate GDF15 expression in 3T3-L1 adipocytes, the latter via FXR but not via TGR5. Among innate immune regulators, only TLR7 activation and hypoxic mediators upregulate whereas STAT3 signaling downregulates GDF15. **Conclusion**: GDF15 expression in human SAT and VAT is correlated to peripheral blood GDF15 concentrations and is regulated by metabolic and innate immune response pathways involved in AT inflammation and metaflammation.

## 1. Introduction

The pleiotropic and secretory protein GDF15 (growth/differentiation factor-15) was originally identified as a transcript (macrophage inhibitory cytokine 1, MIC1) in activated macrophages [1] in 1997. GDF15 belongs to the superfamily of transforming growth factor-beta (TGFβ1) and it is correlated with a broad range of cellular stress responses [2]. Cell stress and cell damage releases GDF15 that travels into the systemic circulation. GDF15 is upregulated by inflammatory and metabolic processes [3] in several cell types such as cardiomyocytes, immune cells, monocytes/macrophages, endothelial cells, and adipocytes of human and murine origin [4]. Due to the latter observation, Ding et al. suggested GDF15 as a novel adipokine [4]. GDF15 has been considered a potential biomarker or even a pathophysiological key factor in metabolic stress activation, obesity, type 2 diabetes, hepatic fibrosis, and weight regulation [5]. Elevated levels of GDF15 seem to be substantially involved in the hyperglycemia-related component of the metabolic syndrome complex and also in obesity during weight loss [5,6]. Recently, we could demonstrate that serum GDF15 concentrations in obese patients undergoing bariatric (metabolic) surgery strongly decline during weight loss and that GDF15 levels are able to function as a marker predicting hepatic fibrosis and type 2 diabetes mellitus [5]. However, detailed adipose tissue gene expression studies in this context are currently lacking. A murine knockout model for GDF15 resulted in obesity and increased food consumption alongside reduced energy expenditure and physical activity [7]. Moreover, the interaction of GDF15 with its hindbrain-located heterodimeric receptor GFRAL (glial cell derived neurotrophic factor receptor alpha-like)-RET (rearranged during transfection) causes weight reduction in diet-induced obesity and in obesity models via diminished food intake [8,9,10] and fat uptake [11].

GDF15 has been established to be involved in nutritional and metabolic stress [12,13,14,15] and is being considered a molecular therapy target [16,17]. It represents a substantial factor in obesity and related morbidities such as T2D [18] and systemic GDF15 quantities have been reported to be elevated in obese individuals [19], albeit this correlation might be subject to a sexual dimorphism [20]. Thus, GDF15 expression in adipocytes and adipose tissue—particularly under the conditions of obesity—are of substantial clinical relevance.

As illustrated within the *Human Protein Atlas* (https://www.proteinatlas.org/ENSG00000130513-GDF15/tissue, accessed on 23 March 2025), GDF15 gene transcription is active in multiple organs with highest mRNA concentrations in the kidney, urinary bladder and choroid plexus. Since GDF15 expression in adipose tissue ranks only in the 10th place, detailed data on expression and regulation in human and murine adipocytes and adipocyte cell lines are important. GDF15 was reported to be expressed in human and murine primary adipocytes as well as in adipocyte cell lines of murine origin [4]. GDF15 is upregulated in murine white and brown adipocytes via stress response signaling [11,21], in human preadipocytes by weight reduction induced by caloric restriction [22], and in human adipocytes by mitochondrial ATP synthesis antagonists [23]. Of particular interest, data from a recent study revealed GDF15 to be induced in murine white adipose tissue and to be involved in mechanisms linking mental stress and lipolysis [24].

Despite accumulating data on the metabolic involvement of GDF15, the exact regulation and detailed expression of GDF15 mRNA in total adipose tissue depots as well as in preadipocytes and fully maturated adipocytes of human and murine origin is poorly described, as with the quantity of secreted protein in obesity. In addition, it is unknown whether human adipocyte cell lines such as SGBS cells are a suitable tool for studying the expression of GDF15. Moreover, factors driving the detrimental processes of “metaflammation” systemically and of “adipoflammation” (adipose inflammation) in visceral adipose tissue [25,26,27] are of potential importance for the regulation of GDF15. Among these factors, metabolic regulators (such as glucose, insulin, or bile acids) as well as innate immune response pathways (such as Toll-like receptors (TLR) and hypoxia) [25,26,27] might be of substantial relevance. More specifically, a potential involvement of certain TLR—TLR2/6, TLR3, TLR4, TLR7, and TLR9, all having been shown to be functionally expressed in adipocytes—and established inflammatory pathways are of particular interest.

Following these considerations, the focus of the current study was to

- Analyze the gene expression profile of GDF15 in the human adipocyte cell line SGBS (Simpson–Golabi–Behmel syndrome) during cellular differentiation in order to determine whether SGBS cells are a suitable in vitro model for studying GDF15;
- Examine the gene expression of GDF15 in human subcutaneous and visceral adipose tissue within a clinical cohort of obese individuals being allocated to bariatric (metabolic) surgery and to correlate these expression data with serum GDF15 protein concentrations and with anthropometric and metabolic factors;
- Investigate the effect of classical TLR ligands, hypoxia, and classical signal transduction components on GDF15 gene expression and protein secretion in murine 3T3-L1 adipocytes.

## 2. Materials and Methods

### 2.1. GDF15 mRNA Expression Analysis by Quantitative PCR

Total RNA was obtained from 3T3-L1 adipocytes, SGBS adipocytes, and human adipose tissue specimens by applying the RNeasy^®^ Mini Kit (Qiagen, Hilden, Germany; catalog number (cat.) 74106). Nucleic acids were extracted from adipose tissue with TRIzol reagent (Thermo Fisher, Braunschweig, Germany; cat. 15596018) and chloroform (Sigma-Aldrich, Deisenhofen, Germany; cat. 288306) prior to subsequent RNA isolation. Synthesis of cDNA complementary to mRNA (QuantiTect Reverse Transcription Kit from Qiagen, Hilden, Germany; cat. 205314) was performed, followed by quantitative real-time PCR (qRT-PCR) (iTaq Universal SYBR Green Supermix for CFX Connect RT-PCR system; Bio-Rad, Munich, Germany; cat. 1725125). Expression levels of target genes were quantified by applying the following primer pairs (forward/reverse sequences):

Murine GAPDH: 5′-TGTCCGTCGTGGATCTGAC-3′/5′-AGGGAGATGCTCAGTGTTGG-3′
Murine GDF15: 5′-CCGAGAGGACTCGAACTCAG-3′/5′-TAAGAACCACCGGGGTGTAG-3′
Human adiponectin: 5′-GACCAGGAAACCACGACTCA-3′/5′-CCTTAGGACCAATAAGACCTGGA-3′
Human GAPDH: 5′-GAGTCCACTGGCGTCTTCAC-3′/5′-CCAGGGGTGCTAAGCAGTT-3′
Human GDF15: 5′-CGGTGAATGGCTCTCAGATG-3′/5′-CAGGTCCTCGTAGCGTTTCC-3′

Oligonucleotides were purchased from Metabion (Martinsried, Germany). Relative cDNA/mRNA quantities of GDF15 were determined by applying the ddC_T_ method, applying GAPDH as a housekeeping parameter for normalization. GAPDH has been established as a reliable reference gene for gene expression analysis in adipocytes in previous studies [28,29]. For human adipose tissue, data on expression levels of GDF15 are given in arbitrary units (GDF15 copy numbers per 1 million of GAPDH copies).

### 2.2. Enzyme-Linked Immunosorbent Assay (ELISA)

GDF15 protein quantities in human blood serum were measured by ELISA (DuoSet Development Kit for human GDF15, Bio-Techne, Minneapolis, MN, USA; cat. DY957). The applied ELISA kit provided a detection range of 7.8–500 pg/mL. Murine GDF15 levels in adipocyte culture supernatants were quantified by applying an ELISA kit specific for the murine protein (Mouse DuoSet Development Kit, Bio-Techne; Minneapolis, MN, USA; cat. DY6385), with a detection range of 7.8–500 pg/mL. The measurements were performed in technical duplicates. In cases of intra-duplicate CV exceeding a deviation of 20%, analysis of the respective samples was repeated.

### 2.3. Cell Culture of 3T3-L1 Adipocytes

For in vitro analysis of GDF15 regulation, the well-established adipocyte model derived from the 3T3-L1 cell-line was applied. Hormonally induced differentiation of 3T3-L1 fibroblast-like cells into mature adipocytes was performed by a previously reported protocol [30] prior to stimulation experiments. Being cultured at 37 °C and 5% CO_2_ in Dulbecco’s Modified Eagle Medium (ATCC, Manassas, VA, USA; cat. 30-2002) supplemented with 10% newborn calf serum (Sigma-Aldrich, Deisenhofen, Germany; cat. N4762), the cells were incubated in DMEM/F12/glutamate medium (PAN-Biotech, Aidenbach, Germany; cat. P04-41250) supplemented with 20 µM 3-isobutyl-methyl-xanthine (IBMX; Serva, Heidelberg, Germany; cat. 26445), 1 µM corticosterone (Sigma-Aldrich; cat. C2505), 100 nM insulin (Sigma-Aldrich; cat. I6634), 200 µM ascorbate (Sigma-Aldrich; cat. A92902), 2 µg/mL transferrin (Sigma-Aldrich; cat. T1428), 5% fetal calf serum (FCS; Sigma-Aldrich; cat. F6765), 1 µM biotin (Sigma-Aldrich; cat. B4639), 17 µM pantothenate (Sigma-Aldrich; cat. P5155), 100 nM rosiglitazone (Sigma Aldrich; cat. R2408), and 300 µg/mL fetuin (Sigma-Aldrich; cat. F3385) for adipocyte differentiation. Characteristic changes in cellular morphology resulting in an adipocyte-like phenotype were evaluated by light-microscopy throughout differentiation [30]. Subsequent experimental approaches were performed exclusively in cells exhibiting a characteristic adipocyte phenotype after differentiation. Prior to stimulation experiments performed with overnight incubation (18 h), mature adipocytes were incubated in serum-free DMEM/F12 medium for 24 h. The following agents were applied in experiments:

- Oligodeoxynucleotides (ODN) class A (5 and 20 µg/mL) and C (10 and 20 µg/mL) (TLR9 agonists; Invivogen, San Diego, CA, USA; cat. tlrl-1585 and tlrl-2395);
- Polyinosinic:polycytidylic acid (poly-IC) (1 and 5 µg/mL; TLR3 agonist; Invivogen; cat. tlrl-pic);
- Macrophage activating lipopeptide 2 (MALP-2) (100 ng/mL; TLR2/6 agonist; Enzo Life Sciences/Axxora, Lörrach, Germany; cat. ALX-162-027);
- Imiquimod (IMQ; 1, 5, and 20 µg/mL) (TLR7 agonist; Invivogen; cat. tlrl-imqs-1);
- BAY11-7085 (5 µM; NF-κB antagonist; Sigma-Aldrich; cat. B5681);
- LY294002 (5 µM; PI3K antagonist; Sigma-Aldrich; cat. 440202);
- S3I-201 (50 µM; STAT3 antagonist; Sigma-Aldrich; cat. 573102);
- L-mimosine (400 µM; HIF1α agonist; Sigma-Aldrich; cat. M0253);
- Acriflavine (2 µM; HIF1α antagonist; Sigma-Aldrich; cat. 01673);
- Ursodeoxycholic acid (UDCA; 100 µM) (Sigma-Aldrich; cat. U5127);
- Obeticholic acid (INT-747) (5 and 20 µM; FXR agonist; Sigma-Aldrich; cat. SML3096);
- S-EMCA (INT-777) (5 and 20 µM; TGR5 agonist; Merck, Darmstadt, Germany; cat. ADVH9B9B3550).

Applied agonist concentrations and treatment conditions in the stimulation experiments were established and validated in preliminary experiments and previous studies [28,29,31,32]. Controlling for unintended effects on cell viability, activity of LDH (lactate dehydrogenase) was measured in cell supernatants following the experiments (Cytotoxicity Detection Kit; Merck; cat. 11644793001). Samples with evidence of cytotoxic effects were excluded from further analysis.

### 2.4. Cell Culture of SGBS Adipocytes

Simpson–Golabi–Behmel syndrome (SGBS) preadipocytes represent a primary human cell line that was previously derived from adipose tissue specimens of an individual with SGBS [33] and were kindly provided by Prof. Martin Wabitsch (University of Ulm, Germany) [34]. These cells are characterized by a strong capacity of differentiation and represent an adequate and well-established human adipocyte in vitro model. The provider’s established protocol was applied for hormonal adipocyte differentiation within 14 days. SGBS cells were cultured in DMEM/F12 (1:1) medium (Invitrogen, Darmstadt, Germany) supplemented with 10% FCS. Upon reaching 70–80% confluence, differentiation of the preadipocytes into mature adipocytes was induced by incubation in serum-free medium supplemented with 10 µg/mL transferrin (Sigma-Aldrich; cat. T2252), 20 nM insulin (Sigma-Aldrich; cat. 10908), 0.2 nM triiodothyronine (Sigma-Aldrich; cat. T6397), and 100 nM cortisol (Sigma-Aldrich; cat. H4001). During the first 4 days, 250 μM isobutylmethylxanthine (IBMX; Serva, Heidelberg, Germany; cat. 26445), 2 μM rosiglitazone (Sigma Aldrich; cat. R2408), and 25 nM dexamethasone (Sigma-Aldrich; cat. D1756) were added to the medium. The differentiation medium was replaced every fourth day and cell morphology of preadipocytes and mature adipocytes was visually controlled by light microscopy. Additionally, functional adipocyte maturation was evaluated by quantification of adiponectin mRNA expression as a late-stage differentiation marker. Exemplarily, adiponectin expression is significantly induced (*p* = 0.001) in differentiated cells (day 11 versus cells during early differentiation at day 4). Fully differentiated adipocytes were incubated in serum-free medium for 24 h prior to stimulation experiments, which were then performed with overnight incubation (18 h). Experimental settings simulating hyperglycemia and hyperinsulinemia including concentrations of glucose (5.6 and 25 mM) and insulin (0.2 and 2 nM) were adjusted in the serum-free medium. LDH activity in the cell supernatants was quantified (Cytotoxicity Detection Kit; Merck; cat. 11644793001) for all experimental samples in order to identify any unintended cytotoxic effects and to exclude impaired samples from further analysis.

### 2.5. Human Adipose Tissue Samples

Tissue specimens were obtained from visceral and subcutaneous adipose tissue of n = 96 obese (mean BMI 53.35 ± 7.03 kg/m^2^; 20 men, 76 women) and deeply characterized patients during bariatric (metabolic) surgery in the context of the *ROBS* (Research in Obesity and Bariatric Surgery) study cohort. *ROBS* is an open-label, non-randomized, monocentric, prospective, and observational (explorative and confirmatory) study of patients routinely allocated to either bariatric surgery (gastric sleeve or Roux-en-Y gastric bypass) or a low-calorie formula diet (LCD) in the tertiary care center at the University Hospital Giessen, Germany. More detailed information on this study has been reported previously [35,36]. Gene expression data from these samples were applied for correlation and subgroup analysis with the patients’ anthropometric and physiological data. The study was approved by the local ethical committee at the University of Giessen, Germany (AZ 101/14). All patients gave their informed consent and were informed about the aim of the study. Data anonymization and privacy policy were accurately applied.

### 2.6. Statistical Analysis

Data were statistically analyzed using the software package SPSS (Version 29.0; IBM, Armonk, NY, USA). Comparison of unrelated groups was performed applying Mann–Whitney U-test (n = 2 groups) or Kruskal–Wallis test (n > 2 groups). Related samples were compared by Wilcoxon test (n = 2 groups) or Fisher test (n > 2 groups). Bonferroni correction was applied in order to correct for multiple comparisons. Group comparisons are graphically presented either as bar diagrams (means ± 2-fold standard error of the mean, SEM) or as box plots. In the box plot graphs, whiskers illustrate interquartile ranges, whereas dots and stars represent outlying values exceeding the interquartile range (IQR; between 25 and 75 percentile) (dots (circles): mild outliers 1.5× to 3× IQR; stars (asterisks): extreme outliers more than 3× IQR). Correlation analysis of numerical variables was performed applying Spearman’s rank correlation coefficient, with data being depicted as scattered plots. In the text and in the table, data are given as means ± 2-fold standard deviation. All in vitro experiments were executed at a minimum of n = 12 replicates. For clinical data, case numbers are depicted in the respective figures and tables. *p* values < 0.05 were considered statistically significant.

## 3. Results

### 3.1. Expression and Regulation of GDF15 mRNA in Human SGBS Adipocytes

**Figure 1:**
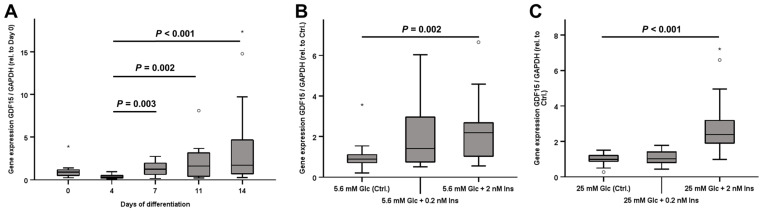
GDF15 mRNA expression by qPCR in human SGBS adipocytes. (**A**): GDF15 gene expression is strongly upregulated during differentiation of SGBS adipocytes over 14 days (n = 15–17). (**B**): Effect of insulin (0.2 nM, 2.0 nM) on GDF15 expression under normoglycemic (5.6 mM glucose) conditions (n = 19–23). (**C**): Effect of insulin (0.2 nM, 2.0 nM) on GDF15 gene expression under hyperglycemic (25 mM glucose) conditions (n = 26–30). Glc, glucose; Ins, insulin.

Since GDF15 expression in SGBS adipocytes has not been investigated so far, we aimed to test whether these cells are suitable for GDF15 research. As depicted in Figure 1A, GDF15 mRNA concentrations are very low in undifferentiated SGBS preadipocytes from day 0 to day 4. GDF15 expression is significantly induced during differentiation over 14 days and in a stepwise manner from day 7 to day 14. However, this effect is not very impressive since the induction is only ~ 2-fold. Insulin at higher doses of 2 nM is able to upregulate GDF15 expression under normoglycemic (5.6 mM glucose) conditions (Figure 1B) and under hyperglycemic (25 mM glucose) conditions (Figure 1C). This effect is considered weak to moderate (~2-fold). Gene expression was not different under hyperglycemic conditions when compared to normoglycemic conditions.

Taken together, basal GDF15 gene expression in the human SGBS cell line is very low and nearly absent. The upregulation during differentiation and upon insulin stimulation is weak and independent of the glucose concentration. Thus, SGBS cells do not seem to represent a suitable cell line for analyzing GDF15 regulation. In contrast, the murine 3T3-L1 adipocyte cell line is an established and easily available in vitro model for studying gene expression on a large scale.

### 3.2. GDF15 mRNA Quantities in Human Visceral and Subcutaneous Adipose Tissue

**Figure 2:**
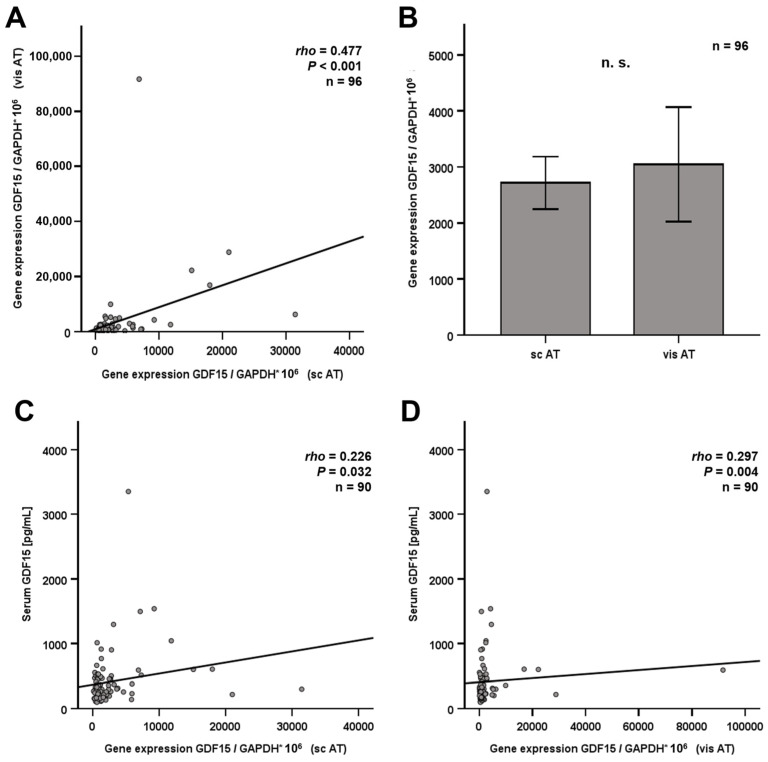
GDF15 mRNA expression by qPCR in human visceral and subcutaneous adipose tissue resected from patients (n = 96) undergoing bariatric surgery. (**A**): GDF15 mRNA is expressed in visceral (vis AT) and subcutaneous adipose tissue (sc AT). GDF15 expression in subcutaneous adipose tissue significantly (*p* = 0.001) and positively (rho = 0.337) correlates with visceral adipose tissue gene expression levels. (**B**): As calculated by the Wilcoxon test for dependent samples, expression levels of GDF15 do not significantly differ between visceral and subcutaneous adipose tissue. (**C**): Correlation of GDF15 mRNA expression in subcutaneous adipose tissue with GDF15 protein levels in peripheral blood serum (measured by ELISA). (**D**): Correlation of GDF15 mRNA concentrations in visceral adipose tissue with GDF15 protein concentrations in peripheral blood serum.

As summarized in Figure 2A, GDF15 mRNA expression is detectable in human visceral and subcutaneous adipose tissue resected from obese bariatric surgery patients (n = 96). The expression levels of GDF15 in both adipose tissue locations correlate positively, while not differing significantly (Figure 2B). The expression of GDF15 in subcutaneous adipose tissue is significantly and positively correlated with the respective GDF15 protein concentrations in peripheral blood serum (Figure 2C) as quantified by ELISA. Similarly, GDF15 expression in visceral adipose tissue correlates significantly with the respective GDF15 protein concentrations in peripheral blood serum (Figure 2D).

In these patients, the GDF15 serum protein concentrations ranged between 96 and 3351 pg/mL (mean ± SD: 415 ± 420 pg/mL). While body weight and body mass index (BMI) show either no or only marginal correlations with GDF15 expression in adipose tissue, the waist-to-hip ratio (WHR) is significantly and positively correlated to GDF15 gene expression (Table 1). Since insulin upregulates GDF15 in vitro (Figure 1B,C), we aimed to test the correlation between serum insulin concentrations with adipose tissue GDF15 mRNA quantities. As shown in Table 1, serum insulin is highly correlated with GDF gene expression mainly in subcutaneous adipose tissue, but also in visceral adipose tissue. In contrast, GDF15 gene expression showed no correlations with serum glucose, glycosylated hemoglobin (HbA_1c_) or C-reactive protein (CRP).

Taken together, GDF15 gene expression is detectable in human visceral and subcutaneous adipose tissue without significant differences regarding quantity. GDF15 mRNA levels in both adipose tissue depots correlate with each other as well as with WHR and insulin. Importantly, GDF15 gene expression in both adipose tissue depots show a significant and positive correlation with the respective serum concentrations of the corresponding GDF15 protein.

Considering the outliers in GDF15 serum concentrations (Figure 2C,D), more detailed subgroup analysis was performed. Of note, comparison tests revealed substantially elevated HbA_1c_ percentage in patients with very high GDF15 levels (>1000 pg/mL) when compared to those with lower GDF15 quantities (6.98% versus 6.12%, *p* = 0.015), whereas metabolic and inflammatory parameters—BMI, insulin, glucose, and CRP—did not significantly differ between both subgroups. Outlying GDF15 mRNA quantities as depicted in Figure 2 were not associated with significant differences regarding the aforementioned parameters.

### 3.3. Effect of Toll-like Receptor (TLR) Agonists on the Regulation of GDF15 Transcription Levels in Murine 3T3-L1 Adipocytes

**Figure 3:**
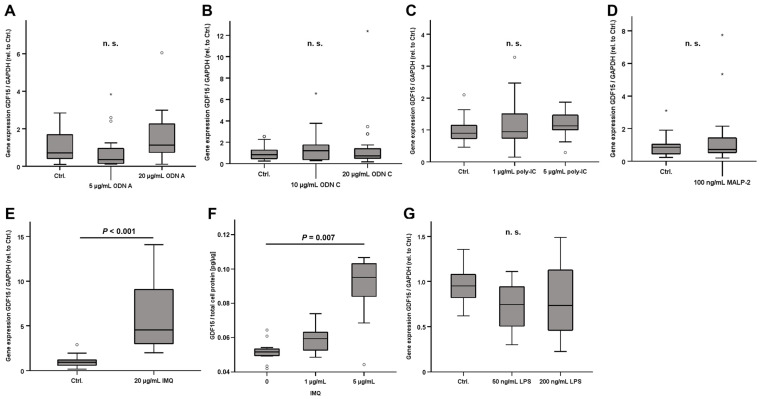
Impact of Toll-like receptor (TLR) agonists on the regulation of GDF15 gene expression in murine 3T3-L1 adipocytes. (**A**): The TLR9 agonistic oligodeoxynucleotide ODN A (5, 20 µg/mL) does not upregulate GDF15 gene expression when compared to unstimulated controls (Ctrl.) (n = 15–21). (**B**): The TLR9 agonistic oligodeoxynucleotide ODN C (15, 20 µg/mL) does not modulate GDF15 gene expression (n = 14–23). (**C**): The TLR3 agonistic Poly I:C (1, 5 µg/mL) does not modify GDF15 gene expression (n = 14–19). (**D**): TLR2/6 agonist MALP-2 (100 ng/mL) does not increase GDF15 gene expression (n = 14–18). (**E**): The TLR7 agonistic imiquimod (IMQ, 20 µg/mL) significantly upregulates GDF15 mRNA expression (n = 23–24). (**F**): The TLR7 agonistic imiquimod (IMQ, 20 µg/mL) significantly upregulates GDF15 protein secretion (n = 12). (**G**): The TLR4 agonistic lipopolysaccharide (LPS; 50, 200 ng/mL) failed to increase GDF15 gene expression (n = 17–18).

The TLR9 agonistic oligodeoxynucleotides ODN A and ODN C do not upregulate GDF15 gene expression (Figure 3A,B). Similarly, neither TLR3 agonistic Poly I:C nor lipopeptide MALP-2 as a TLR2/6 agonist are able to increase GDF15 gene expression (Figure 3C,D). Interestingly, the TLR7 agonistic imiquimod significantly upregulated GDF15 mRNA expression (Figure 3E) as well as GDF15 secretion into adipocyte cell supernatants as measured by ELISA (Figure 3F). The TLR4 ligand lipopolysaccharide (LPS) failed to modify GDF15 gene expression (Figure 3G).

Thus, among all Toll-like receptors known to be involved in adipocyte physiology [37], only TLR7 ligands are able to induce GDF15 gene expression.

### 3.4. Effect of HIF-1α (Hypoxia-Inducible Factor-1α) Modulators and Signal Transduction Inhibitors on the Regulation of GDF15 Transcription and Protein Secretion in 3T3-L1 Adipocytes

**Figure 4:**
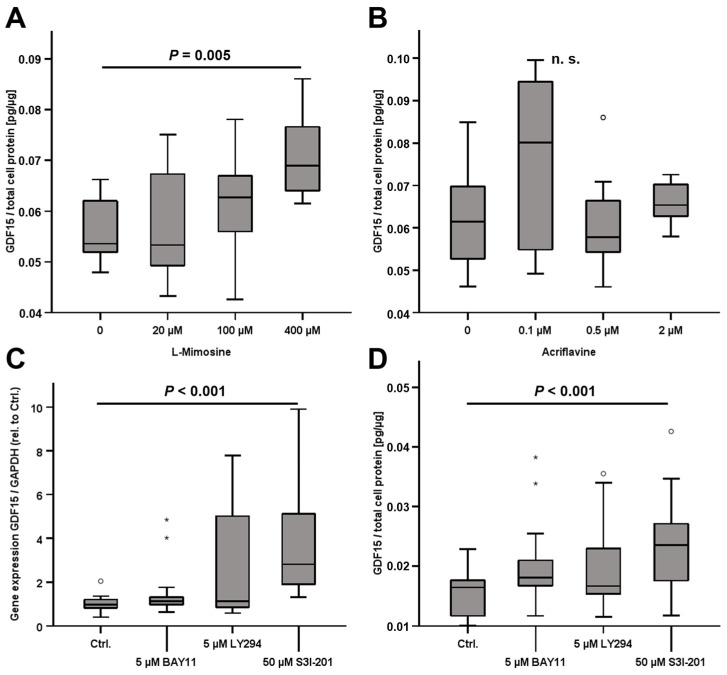
Effects of HIF-1α (hypoxia-inducible factor-1α) modulators and signal transduction inhibitors on the regulation of GDF15 mRNA synthesis and protein secretion in 3T3-L1 adipocytes. (**A**): The HIF-1α agonist L-mimosine (400 µM) (n = 12) significantly upregulates GDF15 protein secretion whereas the HIF-1α inhibitor acriflavine (2 µM) (n = 12) has no effect (**B**). (**C**): Effect of signal transduction inhibitors for NFκB (5 µM BAY11), PI3K (5 µM LY294), and STAT3 (5 µM S3I-201) on GDF15 mRNA expression (n = 16–18). (**D**): Effect of signal transduction inhibitors for NFκB (5 µM BAY11), PI3K (5 µM LY294), and STAT3 (5 µM S3I-201) on GDF15 protein secretion into adipocyte cell supernatants (n = 24).

Since GDF15 is considered a marker of metabolic stress, we hypothesized that hypoxia—a substantial element of metabolic disbalance in obesity-related hypertrophic adipose tissue—might affect GDF15 expression in adipocytes. We therefore investigated a potential involvement of the HIF-1α pathway. As shown in Figure 4A, the HIF-1α agonist L-mimosine was able to increase GDF15 protein secretion into the supernatants of adipocytes whereas the HIF-1α inhibitor acriflavine had no significant effect on protein secretion (Figure 4B). A panel of standard signaling pathway inhibitors for NFκB, PI3K, and STAT3 on basal GDF15 mRNA expression was investigated. We found consistent data regarding mRNA quantities and protein secretion of GDF15 only for STAT3 inhibition. STAT3 inhibition significantly upregulated adipocyte GDF15 transcription levels and protein secretion (Figure 4C,D).

Taken together, among innate immune response activators and pattern recognition pathways, TLR7 and hypoxia are of relevance regarding GDF15 gene regulation. STAT3, described as a mediator of metabolic remodeling and downstream signaling component of adipokines [38] such as leptin, exerts inhibitory effects on GDF15 expression, at least on the regulation of unstimulated, basal GDF15 gene expression.

### 3.5. Effect of Bile Acids, FXR Agonists and TGR5 Agonists on GDF15 mRNA Expression and Protein Secretion in 3T3-L1 Adipocytes

**Figure 5:**
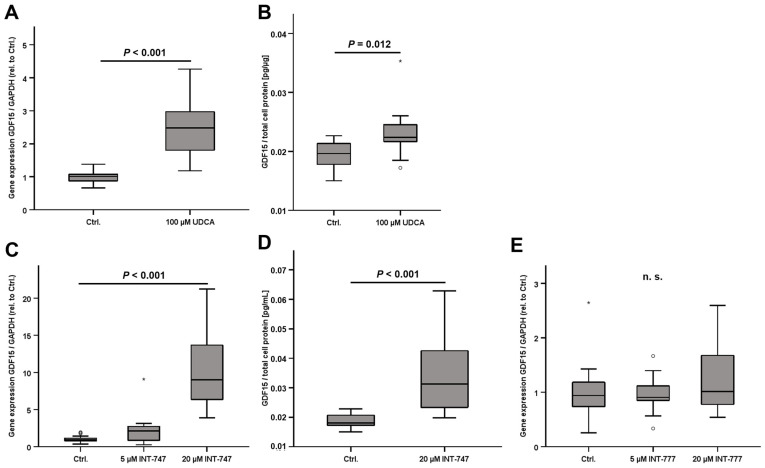
Effects of ursodeoxycholic acid and bile acid receptor agonists on GDF15 mRNA expression and protein secretion in 3T3-L1 adipocytes. (**A**): Ursodeoxycholic acid (UDCA, 100 µM) significantly upregulates GDF15 gene expression (n = 18). (**B**): Effect of UDCA on GDF15 protein secretion into adipocyte cell supernatants, measured by ELISA (n = 12). (**C**,**D**): The FXR receptor agonist obeticholic acid (INT-747: 5, 20 µM) significantly upregulates GDF15 gene expression (n = 20–24) as well as protein secretion (n = 24). (**E**): Missing effect of the TGR5 receptor agonist INT-777 (5, 20 µM) on GDF15 gene expression (n = 21–24).

As presented in Figure 5A, the potent tertiary bile acid ursodeoxycholic acid (UDCA) significantly upregulated GDF15 gene expression and GDF15 secretion (Figure 5B). Examining whether this UDCA effect was mediated by genomic effects via the nuclear bile acid receptor FXR or by non-genomic effects via the cellular transmembrane receptor TGR5, experiments with synthetic and specific receptor agonists were applied. Importantly, the synthetic FXR receptor agonist obeticholic acid (INT-747) significantly upregulated GDF15 gene expression (Figure 5C) as well as protein secretion (Figure 5D). In contrast, the specific TGR5 receptor agonist INT-777 failed to modulate GDF15 gene expression (Figure 5E).

These findings indicate that the potent and prototypic bile acid UDCA [25] upregulates GDF15 gene expression and protein secretion. This effect appears to be mediated exclusively by the nuclear bile acid receptor FXR but not by the bile acid transmembrane receptor TGR5.

## 4. Discussion

Due to the broad expression profile of GDF15 mRNA, a study was needed to address the specific expression profile and regulation of GDF15 in cultured adipocyte and in different adipose tissue locations. The present study confirms and extends the data by Ding et al. [4]. Moreover, it clearly demonstrates the expression profile of GDF15 in human visceral and subcutaneous adipose tissue within a large and well-characterized cohort of obese patients undergoing bariatric (metabolic) surgery. GDF15 expression levels in both adipose tissue depots are positively related to the respective protein concentrations of GDF15 in peripheral blood.

Additionally, our study describes for the first time the expression profile of GDF15 in the human adipocyte cell line SGBS. Due to the very low expression quantities, this cell line might not be suitable in contrast to 3T3-L1 adipocytes. Of note, contradictory results have recently been reported by Kim et al. for human adipose-derived stem cells [39]. This discrepancy might be based on specific characteristics of the differing cellular models and should be addressed by future studies. Compared to SGBS cells, 3T3-L1 adipocytes represent a more established and easily available cell model for extensive stimulation experiments on a large scale. Moreover, these cells are more cost effective in culture and can be differentiated in a shorter period (7 days vs. 14 days). Thus, we used differentiated 3T3-L1 adipocytes for extensive and subsequent stimulation experiments.

Most recently, we could demonstrate an insulin-mediated upregulation of GDF15 secretion in murine 3T3-L1 adipocytes cultured in normo- and hyperglycemic conditions [40]. In contrast, GDF15 expression was not different under hyperglycemic vs. normoglycemic conditions in vitro. While we did not observe any significant correlations of GDF15 mRNA concentrations in human adipose tissue with serum levels of glucose and HbA_1c_ within the examined obesity cohort, elevated HbA_1c_ percentage—considered a reliable long-term marker for blood glucose levels—was found in individuals with exceptionally high circulating GDF15 concentrations (>1000 pg/mL). Taken together, hyperinsulinemia seems to represent a metabolic regulator of GDF15 whereas a putative association with hyperglycemia remains debatable.

Obesity can successfully be treated by low-calorie diets, bariatric (metabolic) surgery, and GLP-1 agonists. Most recently, we observed the fascinating role bile acids could play in adipocyte physiology and in the context of weight loss being achieved by either bariatric surgery or low-calorie diets [25,36]. Bile acids are able to act as hormone-like molecules [41] on adipocytes by exerting either genomic effects (via nuclear receptor FXR) or non-genomic effects (via Takeda G-protein coupled transmembrane receptor TGR5). We could demonstrate for the first time that the potent and tertiary bile acid ursodeoxycholic acid (UDCA)—that has been clinically used for treating fatty liver disease or cholestatic liver disease—upregulates GDF15 expression and secretion in adipocytes. This effect of UDCA can be attributed to genomic effects mediated by FXR since only synthetic FXR agonists but not TGR5 agonists were able to reproduce the effects of UDCA. This observation is only of descriptive nature in the present study, but it might have a highly important and clinical potential as a drug target for the treatment of pathologies within the metabolic syndrome and should be investigated by future and detailed studies.

Innate immune response mediators and pattern recognition receptors are critically involved in adipoflammation and metaflammation, especially Toll-like receptors [28,37]. Therefore, we screened a broad panel of Toll-like receptor ligands (TLR2, -3, -4, -6, -7, -9) for their capacity to regulate GDF15 expression. Recently, we could demonstrate that endosomal TLR7 (recognizing single-stranded RNA) is expressed in adipocytes and that its activation modulates adipokine expression and glucose transporter-4 expression [32]. Interestingly, TLR7 activation upregulated GDF15 transcription and secretion in the present study whereas activation of all the other TLRs failed to exert any effects.

Within the examined cohort of severely obese individuals, adipose tissue GDF15 expression did not significantly correlate with circulating CRP quantities as a general systemic inflammation marker. Since hepatic-derived CRP represents a relatively crude and unspecific inflammation marker, the results of the present study are primarily of descriptive and exploratory nature and have to be interpreted with caution regarding this issue. Future measurement of pro-inflammatory cytokines such as IL-1, IL-6 and TNF and testing of their putative association to GDF15 expression would help to improve the impact of this study. Additionally, future approaches should perform an even more detailed investigation of a wider spectrum of inflammatory parameters, including the quantification of mRNA levels within extracellular vesicles (small EV fractionation from plasma/serum and subsequent quantitative PCR).

In obesity, hypoxia occurs in visceral adipose tissue leading to a pro-inflammatory milieu that triggers local insulin resistance, adipocyte necrosis, monocyte infiltration, adipokine secretion, and extracellular matrix fibrosis [42,43]. During these processes of adipoflammation and metaflammation [27,44], HIF1α plays an important role. We could demonstrate that the HIF1α activator L-mimosin upregulates GDF15 secretion, indicating that hypoxia-related pathways and subsequent HIF1α activation exert stimulatory mechanisms on GDF15 regulation. Since GDF15 can generally be regarded as a protein upregulated by cellular stress mechanisms, this observation seems reasonable. This should be further investigated by molecular and mechanistic studies. The connection between HIF1α and GDF15 represents a novel aspect in adipocyte physiology. There is one study [45] that described a differential regulation in the special context of hyperoxia and HIF1α in human umbilical vein cells. Future experiments should check the direct effects of hypoxia on GDF15 by using hypoxia chambers as a more physiologically relevant procedure.

The most striking limitation of this study is based on its primarily descriptive and explorative nature which limits biological relevance and interpretability of the findings. Importantly, a screening approach was performed in order to identify inflammatory pathways potentially involved in the regulation of adipocyte GDF15 expression. The aim was to provide initial data as a basis for subsequent, more specific elaboration. Therefore, the present results should be clarified by mechanistic and causative investigations. In particular, the regulation of GDF15 expression in adipocytes by pro-inflammatory cytokines should be addressed. Moreover, the serum levels of the corresponding GDF15 protein should be correlated with systemic inflammation markers other than CRP. The investigation of human cell lines, murine cell lines, and human adipose tissue obtained from different depots by using a large study cohort represents an advantage. In vitro experiments focused mainly on murine 3T3-L1 adipocytes as a very well characterized and established cell culture model. While these cells have been proven to represent an adequate model for intracellular studies on adipocyte biology, transferability of the observed effects to the human system has to be considered with caution and requires evaluation in future experiments using human adipocytes. Since GDF15 can be regarded as a metabolically protective protein, bile acids and TLR7 agonists should be investigated as prospective novel drug targets for the treatment of obesity and metabolic syndrome. In contrast, the upregulation of GDF15 by hypoxic mediators might represent a counter-regulatory mechanism in visceral obesity.

### Summary

GDF15 expression is induced in human adipocyte differentiation and is present in human adipose tissue of subcutaneous and visceral origin. GDF15 mRNA quantities in visceral and subcutaneous adipose tissue correlate with each other and with GDF15 concentrations in peripheral blood serum of obese patients. Among metabolic factors, only insulin but not glucose induces GDF15 in vitro. Moreover, GDF15 expression in adipose tissue positively correlates with serum insulin concentrations. When compared to murine 3T3-L1 adipocytes, human SGBS adipocytes do not seem to provide a suitable in vitro model for gene expression studies on a large scale due to the very low GDF15 gene expression levels. Bile acids such as ursodeoxycholic acid upregulate GDF15 expression via FXR but not via TGR5. Among innate immune regulators and Toll-like receptors, TLR7 activation and HIF1α activation upregulate GDF15. However, the current study is mainly of descriptive and explorative nature and future mechanistic and causative investigations are warranted, especially in the context of inflammatory parameters.

## 5. Conclusions

GDF15 gene expression levels in human adipose tissue correlate to its protein concentrations in serum. In vitro, adipocyte GDF15 expression is regulated by metabolic and innate immune response pathways being involved in adipose tissue inflammation and metaflammation.

## Figures and Tables

**Table 1:** Correlation between anthropometric and clinical chemistry parameters with GDF15 mRNA concentrations in visceral and subcutaneous adipose tissue of obese patients (n = 96). Statistically significant correlations are highlighted in bold. BMI, body mass index; CRP, C-reactive protein. WHR, waist-hip ratio.

Spearman Correlation Analysis	GDF15 Gene Expression Visceral Adipose Tissue		GDF15 Gene Expression Subcutaneous Adipose Tissue	
	*rho*	*p*	*rho*	*p*
Weight [kg] (153.85 ± 27.95)	0.046	0.656	−0.006	0.950
BMI [kg/m^2^] (53.35 ± 7.03)	−0.137	0.184	−0.229	0.025
WHR (n = 82) (0.94 ± 0.12)	**0.251**	**0.023**	**0.277**	**0.012**
Age [years] (39.81 ± 11.62)	0.256	0.012	0.171	0.095
Serum glucose [mg/dL]	0.145	0.171	0.180	0.090
Serum insulin [mU/L]	**0.250**	**0.037**	**0.407**	**<0.001**
Serum HbA_1c_ [%]	0.145	0.196	−0.025	0.827
Serum triglycerides [mg/dL]	0.218	0.047	0.206	0.062
Serum CRP [mg/dL]	−0.122	0.243	−0.119	0.255

## Data Availability

The original contributions presented in this study are included in the article. Further inquiries can be directed to the corresponding author.
